# Application of the QuEChERS procedure for analysis of Δ^9^-tetrahydrocannabinol and its metabolites in authentic whole blood samples by GC–MS/MS

**DOI:** 10.1007/s11419-018-0419-8

**Published:** 2018-05-07

**Authors:** Michal P. Dybowski, Andrzej L. Dawidowicz

**Affiliations:** Faculty of Chemistry, Maria Curie Sklodowska University, Pl. Marii Curie Sklodowskiej 3, 20-031 Lublin, Poland

**Keywords:** QuEChERS, THC, 11-OH-THC, 11-COOH-THC, Whole blood analysis, GC–MS/MS

## Abstract

**Purpose:**

Analysis of drugs and their metabolites in biofluids usually demands the application of sample preparation methods that allow for full isolation of analyzed substances from the matrix. The purpose of this study was to develop a method using the QuEChERS procedure for analysis of Δ^9^-tetrahydrocannabinol (THC), 11-hydroxy-Δ^9^-tetrahydrocannabinol (11-OH-THC) and 11-nor-9-carboxy-Δ^9^-tetrahydrocannabinol (11-COOH-THC).

**Methods:**

THC, 11-OH-THC and 11-COOH-THC were quantified in whole blood samples using QuEChERS extraction and gas chromatography–tandem mass spectrometry.

**Results:**

The described method is characterized by good linearity, very low detection limits and satisfactory inter- and intraday precisions for THC, 11-OH-THC and 11-COOH-THC. The applicability of the procedure was confirmed using authentic whole blood samples collected from 30 persons suspected of driving under the influence of drugs.

**Conclusions:**

The application of QuEChERS extraction described herein is a simple and convenient method for the routine analysis of THC, 11-OH-THC and 11-COOH-THC in whole blood samples from living and deceased humans. To our knowledge, this paper is the first academic report describing the QuEChERS extraction of THC and its metabolites from whole blood specimens.

## Introduction

The screening of human biological fluids (whole blood, plasma, serum and urine) for the presence of Δ^9^-tetrahydrocannabinol (THC), the main active compound in cannabis, and two of its metabolites, 11-hydroxy-Δ^9^-tetrahydrocannabinol (11-OH-THC) and 11-nor-9-carboxy-Δ^9^-tetrahydrocannabinol (11-COOH-THC), is common in both forensic and clinical contexts—for example, in tests for driving under the influence of drugs (DUID) [1–4]. The analysis of drugs and their metabolites in biofluids usually demands the application of a sample preparation method that allows for full isolation of the analyzed substances from the matrices. For this purpose, most approaches involve liquid-liquid extraction (LLE) or solid-phase extraction (SPE). Each of the mentioned techniques is known to have advantages and disadvantages [5–8]. In 2003, Anastassiades et al. [9] developed a new sample preparation technique,“QuEChERS” (a portmanteau word formed from “quick, easy, cheap, effective, rugged and safe”). In fact, the technique combines two extraction processes: classical sample extraction (liquid-liquid or liquid-solid extraction), most frequently performed with acetonitrile in the presence of inorganic salts; and extract purification via a dispersive solid-phase extraction process using different sorbents. Originally, QuEChERS was used for determination of various pesticides in plants and foods [9–13]. Due to its simplicity, high flexibility, low solvent usage and waste minimization, the technique has been continuously modified, becoming increasingly popular as a sample preparation method in analytical procedures for an ever-increasing number of compounds in different matrices, including drugs in biological fluids [14–17].

However, to the best of our knowledge, a detailed method for the isolation of THC and its metabolites by the QuEChERS procedure has not been reported in an academic context, except for brief application notes provided by commercial manufacturers [18, 19]. In the present study, we have established a detailed method for the quantitative analysis of THC, 11-OH-THC and 11-COOH-THC that employs the QuEChERS procedure followed by gas chromatography–tandem mass spectrometry (GC–MS/MS). The method has been applied to the analysis of the compounds in authentic blood specimens collected from 30 DUID cases.

## Materials and methods

### Materials and chemicals

Acetonitrile and methanol (LC/MS grade), anhydrous magnesium sulfate (99.5% powder; MgSO_4_) and sodium chloride were purchased from Merck (Darmstadt, Germany); the Sepra C18-E sorbent (50 µm, 65 Å) used in the QuEChERS process was obtained from Phenomenex (Torrance, CA, USA).

The standards (certified reference materials) of THC (1.0 mg/mL in methanol T-005 Cerilliant), THC-*d*_3_ (1.0 mg/mL in methanol T-011 Cerilliant), 11-OH-THC (1.0 mg/mL in methanol H-027 Cerilliant), 11-OH-THC-*d*_3_ (100 µg/mL in methanol H-041 Cerilliant), 11-COOH-THC (1.0 mg/mL in methanol T-010 Cerilliant) and 11-COOH-THC-*d*_3_ (100 µg/mL in methanol T-004 Cerilliant) were acquired from Sigma-Aldrich (St. Louis, MO, USA). Deionized water was purified on a Milli-Q system (MilliporeSigma, Bedford, MA, USA). Working solutions were prepared in methanol. They were all kept under stable conditions at − 20 °C.

A standard mixture was prepared from individual stock standard solutions to obtain a concentration of 1 μg/mL in acetonitrile. All other working solutions for determining calibration curves, recovery percentages, and limits of detection were prepared by diluting the standard mixture in acetonitrile or in a blank blood sample.

### Collection and storage of blood samples

Blood samples for testing were collected by a qualified person. All the samples were collected from 30 drivers suspected of DUID with positive cannabinoid test results on a Dräger DrugTest 5000 tester (Dräger, Lübeck, Germany). The blood samples (2 × 5 mL) were collected using a single closed system containing an S-Monovette coagulation activator, according to the manufacturer instructions (Sarstedt AG, Nümbrecht, Germany), and thoroughly mixed in order to maintain their homogeneity. Blank whole blood samples for the optimization process, calibration and validation were collected from volunteers with negative saliva cannabinoid test results on the Dräger DrugTest 5000. The biological material was stored in sealed sterile containers at − 20 °C (± 2 °C) before undergoing the QuEChERS procedure.

### QuEChERS procedure and its optimization

The QuEChERS procedure that was applied involves the following six steps:

Step 1: Mechanical mixing of blood sample containing 10 μL of an internal standard solution with appropriate amounts of MgSO_4_ and NaCl for 1 min
Step 2: Addition of a known volume of acetonitrile to the obtained mixture and its extraction for 5 min using a vortex mixer
Step 3: Centrifugation for 10 min (13,750 rpm) at room temperature to enable the separation of the acetonitrile phase and its collection (about 600 μL when 650 μL of acetonitrile was used)
Step 4: Addition of C-18 sorbent to the collected acetonitrile supernatant, with mechanical mixing of the obtained suspension and centrifugation to facilitate sedimentation of the solid C-18 sorbent
Step 5: Evaporation of the purified supernatant (about 550 μL) to dryness under a stream of nitrogen at room temperature
Step 6: Derivatization of the dried sample. The dried residue was reconstituted with 650 μL of derivatization mixture consisting of hexamethyldisilazane (HMDS)/trimethylchlorosilane (TMCS)/pyridine (1.5:1:1.5 v/v; Sigma-Aldrich) and heated at 40 °C for 30 min. The resulting trimethylsilyl (TMS) derivatives were cooled, centrifuged at 3000 *g* for 5 min, and transferred to autosampler vials for GC–MS/MS analysis;

To determine the optimal QuEChERS conditions for the quantification of THC, 11-OH-THC and 11-COOH-THC in blood, the effects of the following amounts of NaCl, MgSO_4_, acetonitrile, C-18 sorbent and blood sample on the recoveries of the above analytes were examined.

1. NaCl (50, 60, 70, 80 or 100 mg), with mass/volume of the other QuEChERS components: blood sample 350 mg; MgSO_4_ 150 mg; acetonitrile 650 µL; and C-18 sorbents 12.5 mg
2. MgSO_4_ (150, 200, 280, 350 or 400 mg), with mass/volume of the other QuEChERS components: blood sample 350 mg; NaCl 80 mg; acetonitrile 650 µL; and C-18 sorbents 12.5 mg
3. Acetonitrile (350, 500, 650, 700 or 750 μL), with mass/volume of the other QuEChERS components: blood sample 350 mg; NaCl 80 mg; MgSO_4_ 150 mg; and C-18 sorbents 12.5 mg
4. C-18 sorbent (10, 12.5, 15, 20 or 25 mg), with mass/volume of the other QuEChERS components: blood sample 350 mg; NaCl 80 mg; acetonitrile 650 µL; and MgSO_4_ 150 mg
5. Blood sample mass (350, 500, 650, 700 and 750 mg), with mass/volume of the other QuEChERS components: NaCl 80 mg; MgSO_4_ 150 mg; acetonitrile 650 µL; and C-18 sorbents 12.5 mg

### Instrumental analysis

For qualification and quantification of the QuEChERS extracts, a gas chromatograph with a tandem mass spectrometer detector (GCMS-TQ8040; Shimadzu, Kyoto, Japan) equipped with a Zebron ZB5-MSi fused-silica capillary column (30 m × 0.25 mm i.d., 0.25 µm film thickness; Phenomenex) was used. Helium (grade 5.0) was used as the carrier gas, and argon (grade 5.0) was used as the collision gas. Column flow was 1.56 mL/min, and 1 µL of the sample was injected using an AOC-20i/s autosampler (Shimadzu). The injector was set to high-pressure mode (250.0 kPa for 1.5 min; column flow at initial temperature was 4.90 mL/min) at 320 °C. The following temperature program was applied:

The oven temperature was held at 60 °C for 2 min and was subsequently increased linearly at a rate of 10 °C/min to 320 °C, where it was held for 15 min.

The mass spectrometer was operated in multiple reaction monitoring (MRM) mode using electron ionization (EI) at 70 eV and an ion source temperature of 220 °C.

Blank whole blood samples were analyzed to confirm the absence of interference peaks at the retention times of the TMS derivatives of THC, 11-OH-THC, 11-COOH-THC and the internal standards of THC-*d*_3_, 11-OH-THC-*d*_3_ and 11-COOH-THC-*d*_3_.

### Method validation and statistical analysis

The method was validated in terms of linearity, the limit of detection (LOD), the limit of quantification (LOQ) and the intraday and interday precision and accuracy measurements. To evaluate the method linearity, five replicated analytical procedures were performed for each examined concentration level. The peak areas were used for the quantification of the calibration curves for all analytes. In order to estimate the LOD and the LOQ, the derivatized extracts from blood samples spiked with the analytical reference standards were injected. The LOD and LOQ were considered to be signal-to-noise ratios equal to 3 and 10, respectively.

The intra- and interday precisions and accuracies were evaluated by statistical analysis of the quantitative results (obtained on the same day and on three different days) for five independent samples containing test compounds (25 ng/mL).

Recovery levels were investigated using blank blood samples spiked with the reference standards of the test compounds at five different concentration levels (1, 5, 10, 15 and 25 ng/mL). They were calculated as the percentage of the analyte response after sample work-up compared to that of a solution containing the analyte at a concentration corresponding to 100% recovery [20]. In order to determine whether there was a significant difference between the recovery percentages at individual analyte concentration levels, a one-way analysis of variance (ANOVA) test was performed.

The linearity of the assay was calculated by the least squares method and expressed as the coefficient of determination (*R*^2^). Calibration plots were prepared using the blank blood samples spiked with the target analytes and internal standards at concentration levels of 0.1, 0.5, 1, 5, 10, 25 and 50 ng/g for THC, 1, 2.5, 5, 10, 25 and 50 ng/g for 11-OH-THC and 1, 2.5, 5, 10, 25 and 50 ng/g for 11-COOH-THC. The solutions were prepared in triplicate.

In order to evaluate possible matrix effects on the analytical signals, the slopes of the calibration graph obtained by the external calibration method were compared to those obtained by the quality control calibration. This was performed by spiking the blood samples with the internal standard solutions of THC-*d*_3_, 11-OH-THC-*d*_3_ and 11-COOH-THC-*d*_3_ at concentration levels similar to those of the sample. The results were compared using Student’s *t* test.

The specificity of the method was evaluated by analyzing the blank whole blood samples from five different volunteers. The samples were tested for the absence of THC and its metabolites.

## Results and discussion

**Fig. 1:**
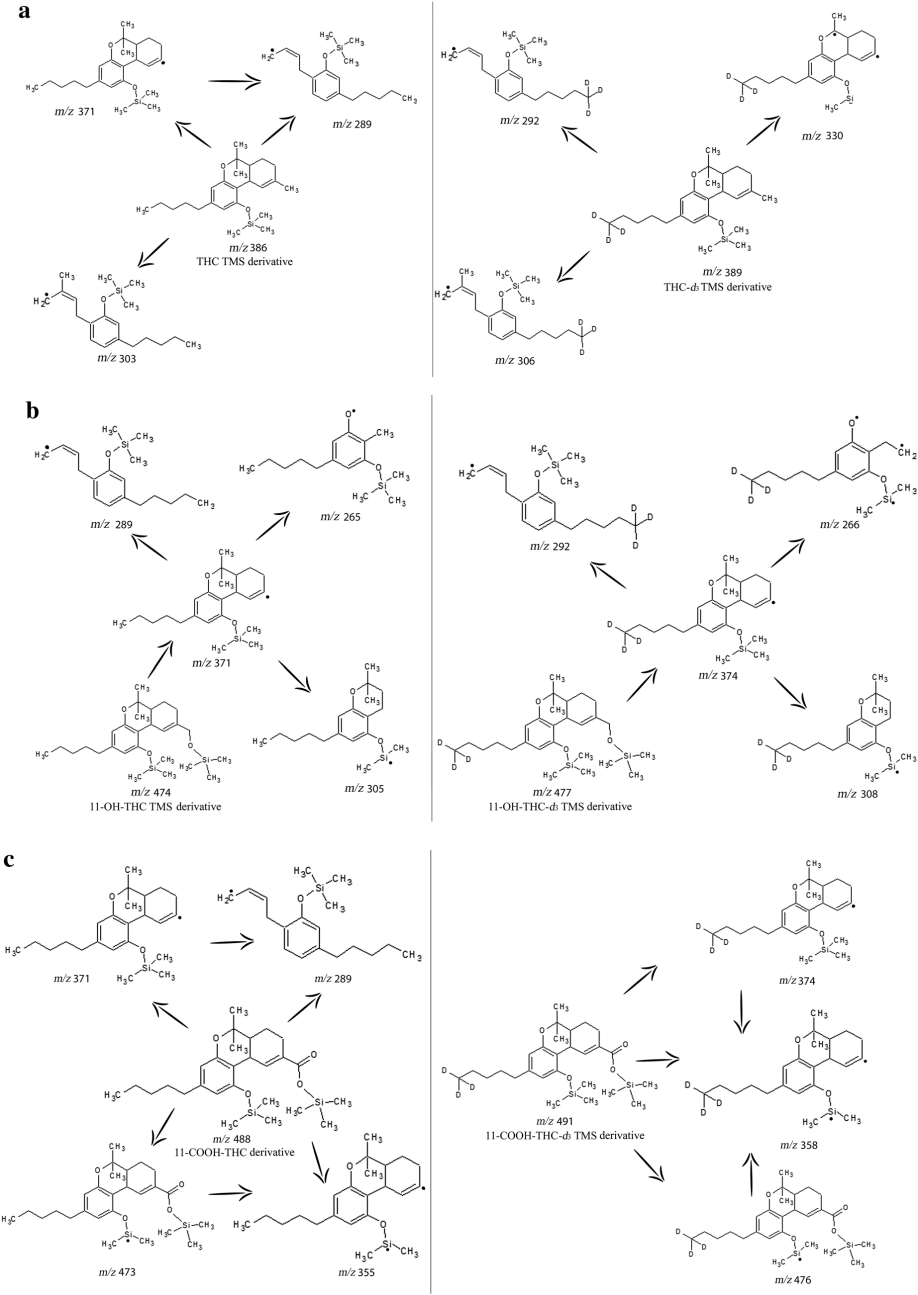
Chemical structures and probable fragmentation pathways of the trimethylsilyl (TMS) derivatives of **a** Δ^9^-tetrahydrocannabinol (THC), THC-*d*_3_, **b** 11-hydroxy-Δ^9^-tetrahydrocannabinol (11-OH-THC), 11-OH-THC-*d*_3_, **c** 11-nor-9-carboxy-Δ^9^-tetrahydrocannabinol (11-COOH-THC), and 11-COOH-THC-*d*_3_

The chemical structures and fragmentation pathways of the TMS derivatives of THC, 11-OH-THC and 11-COOH-THC are shown in Fig. 1a–c. Although the presented fragmentation pathways were essentially based on GC–MS/MS fragmentation data provided by Weller et al. [21], the optimal conditions for our instrumentation were identified for each compound in our laboratory. The MRM transitions and optimal collision energies of the examined compounds are collected in Table 1.

**Table 1:** MRM transitions and collision voltages of THC, THC-*d*_3_, 11-OH-THC, 11-OH-THC-*d*_3_, 11-COOH-THC and 11-COOH-THC-*d*_3_ for gas chromatography–tandem mass spectrometry (GC–MS/MS)

Compound	Retention time (min)	Qualitative MRM transition (mass > product mass)	Quantitative MRM transition (mass > product mass)	Collision voltage (eV)
THC	26.62	371 > 289	386 > 289	12
		386 > 289		
		386 > 303		
THC-*d*_3_	27.12	389 > 306	389 > 292	12
		389 > 292		
		389 > 330		
11-OH-THC	29.31	371 > 289	371 > 265	10
		371 > 265		
		371 > 305		
11-OH-THC-*d*_3_	29.62	374 > 266	374 > 292	10
		374 > 292		
		374 > 308		
11-COOH-THC	31.98	473 > 355	473 > 355	15
		371 > 289		
		488 > 371		
11-COOH-THC-*d*_3_	32.28	374 > 358	374 > 358	15
		476 > 358		
		491 > 374		

*MRM* multiple reaction monitoring, *THC* Δ^9^-tetrahydrocannabinol, *11*-*OH*-*THC* 11-hydroxy-Δ^9^-tetrahydrocannabinol, *11*-*COOH*-*THC* 11-nor-9-carboxy-Δ^9^-tetrahydrocannabinol

**Fig. 2:**
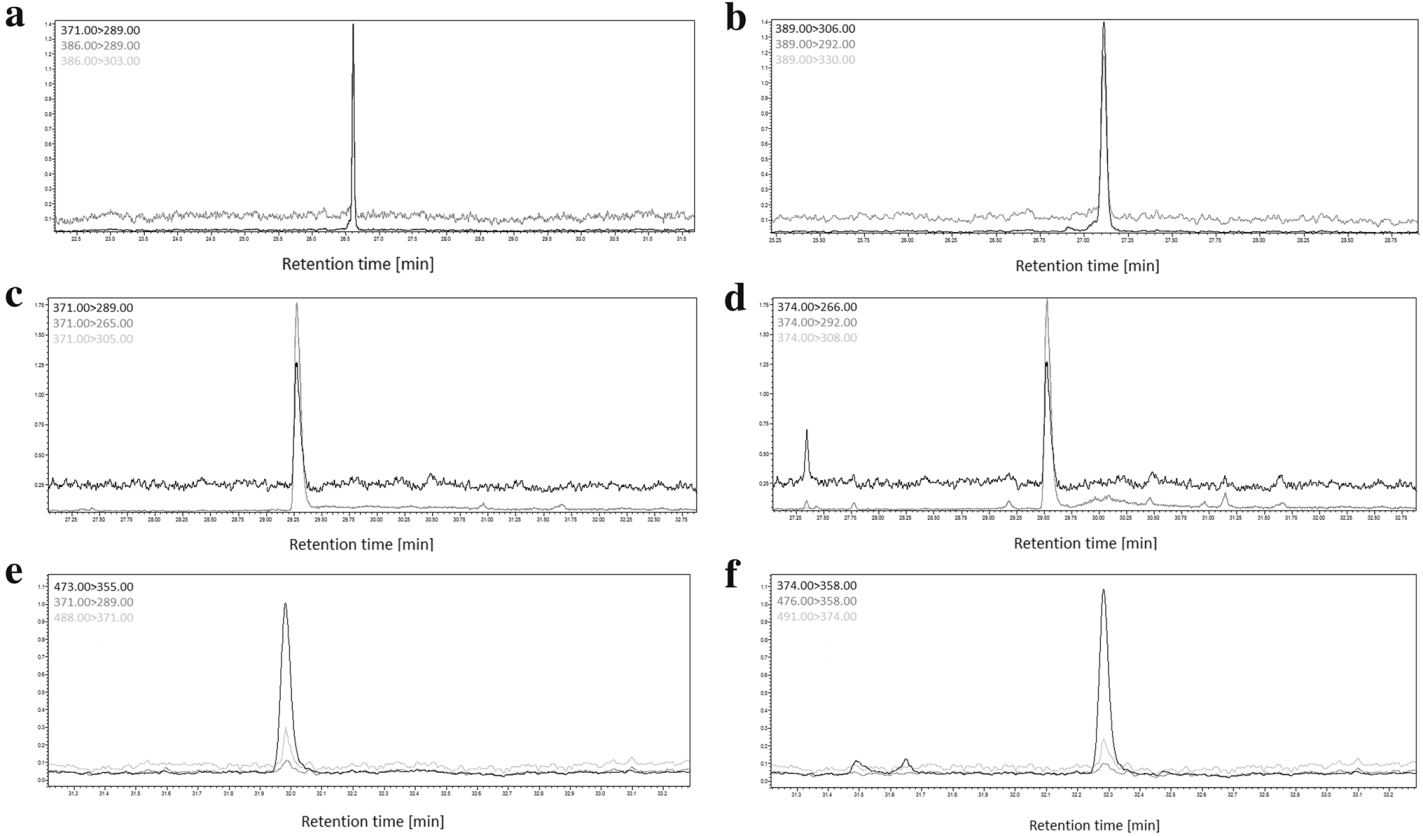
Multiple reaction monitoring chromatograms showing peaks of **a** THC, **b** THC-*d*_3_, **c** 11-OH-THC, **d** 11-OH-THC-*d*_3_, **e** 11-COOH-THC, **f** 11-COOH-THC-*d*_3_, for a QuEChERS extract of an example of actual blood specimens

The exemplary MRM chromatograms of a blank blood sample spiked with THC, 11-OH-THC, 11-COOH-THC (15 ng/g) and deuterated derivatives of these compounds (used as three independent internal standards) following the QuEChERS sample preparation procedure are presented in Fig. 2. Based on the data obtained, the applied GC–MS/MS conditions are acceptable for both the qualitative and quantitative analysis of THC and its two metabolites in whole blood samples.

**Fig. 3:**
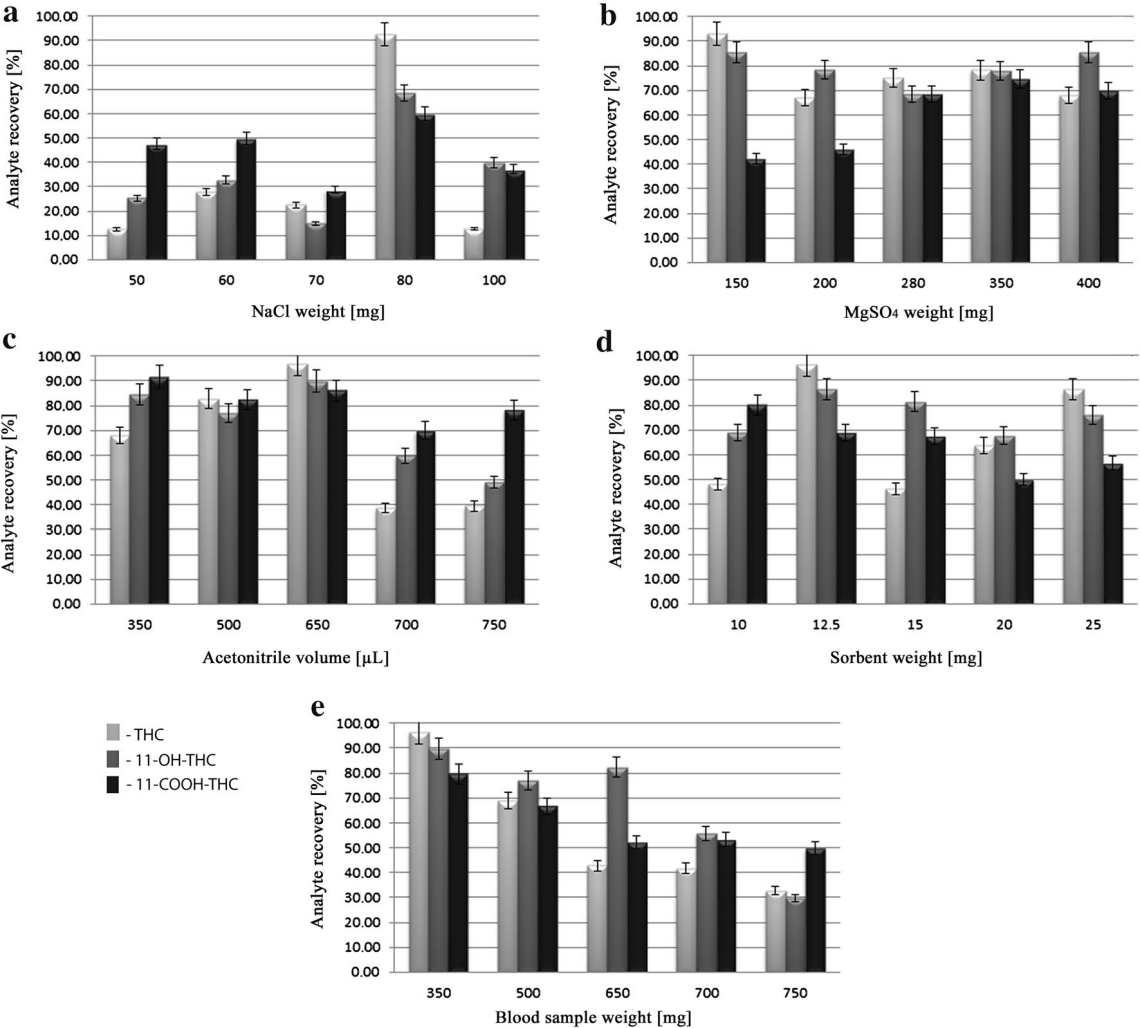
Effects of QuEChERS variables on the recovery rates of THC, 11-OH-THC and 11-COOH-THC. **a** NaCl, **b** MgSO_4_, **c** acetonitrile, **d** C-18 sorbent, and **e** whole blood amounts

The characteristic feature of QuEChERS is the use of inorganic salts in the extraction step and solid C-18 sorbent in the sample purification step. The added inorganic salt decreases the mutual solubility of acetonitrile and water and increases recovery of the analyte. Hence, optimization of the QuEChERS process should account for the effects of the NaCl and MgSO_4_ concentrations on analyte recoveries. A similar requirement concerns the influence of other QuEChERS variables—the amount of acetonitrile and solid C-18 sorbent—on the recoveries. The influence of the amounts of NaCl, MgSO_4_, acetonitrile and C-18 sorbent used in the QuEChERS procedure on the recoveries of THC, 11-OH-THC and 11-COOH-THC in a 350 mg blood sample is shown in Fig. 3a–d. The effect of each factor on the recovery of individual analyte was determined by holding the other QuEChERS variables constant (see “QuEChERS procedure” section), and each factor demonstrated a significant influence on the recoveries of the examined analytes. In addition, the influence of each factor on analyte recovery varied when the other QuEChERS variables were held at different but constant values. The estimation of optimal QuEChERS conditions for individual analytes, especially when attempted for each analyte simultaneously, is difficult. Hence, the application of the QuEChERS procedure for the estimation of THC, 11-OH-THC and 11-COOH-THC in blood required the use of individual deuterated internal standards. This conclusion was confirmed by Fig. 3e, which displays the influence of blood sample quantities on the recoveries of THC and its metabolites. Therefore, we fixed the variables as follows: NaCl 80 mg; MgSO_4_ 150 mg; acetonitrile 650 µL and C-18 sorbent 12.5 mg for a 350 mg blood sample.

In order to estimate analytical utility of the described method, its validation procedure was performed. The results of the validation experiments are gathered in Table 2, and they show that the method is characterized by good linearity, very low detection limits and satisfactory inter- and intraday precisions for THC, 11-OH-THC and 11-COOH-THC.

**Table 2:** Results of validation for the present method

Tested parameter	THC	11-OH-THC	11-COOH-THC
Linearity (*R*^2^)	0.9986	0.9963	0.9971
Intraday precision (% RSD)	3.76	6.26	5.99
Interday precision (% RSD)	4.21	6.79	5.64
Intraday accuracy (%)	98.9	94.2	97.5
Interday accuracy (%)	97.2	102	96.7
LOD (ng/g)	0.011	0.13	0.08
LOQ (ng/g)	0.033	0.43	0.27
Recovery	Recovery percentages, estimated using optimal QuEChERS conditions, of the three analytes spiked into whole blood at 25 ng/g were more than 55%—see Fig. 3		
Matrix effect	No significant differences were found between the slopes. The results led to the conclusion that the presented method was not subjected to any matrix effect		
Selectivity	Absence of peaks of the examined analytes and/or their significant interference on chromatograms confirms the high selectivity of the described method		

*R*^*2*^ coefficient of determination, *RSD* relative standard deviation, *LOD* limit of detection, *LOQ* limit of quantification

To demonstrate the practical applicability of the proposed method, Table 3 presents THC, 11-OH-THC and 11-COOH-THC concentrations estimated for the whole blood samples collected from 30 drivers charged with DUID (positive cannabinoid test results on the Dräger DrugTest saliva tester).

**Table 3:** Analytical results of THC; 11-OH-THC and 11-COOH-THC concentrations in whole blood specimens collected from 30 drivers suspected of driving under the influence of drugs using QuEChERS and GC–MS/MS

Case no.	Age (years)	Sex	Concentration of analyte (ng/g ± SD)		
			THC	11-OH-THC	11-COOH-THC
1	31	M	2.67 ± 0.13	0.74 ± 0.06	16.2 ± 0.99
2	14	M	3.01 ± 0.14	1.11 ± 0.08	9.01 ± 0.55
3	27	M	1.57 ± 0.08	0.57 ± 0.04	0.69 ± 0.04
4	28	M	0.69 ± 0.03	< LOQ	2.21 ± 0.14
5	23	M	0.07 ± 0.002	< LOQ	0.58 ± 0.04
6	24	M	1.49 ± 0.07	3.21 ± 0.24	10.4 ± 0.63
7	32	M	4.02 ± 0.20	5.16 ± 0.39	12.4 ± 0.76
8	23	M	3.16 ± 0.15	0.45 ± 0.03	1.10 ± 0.07
9	24	M	2.19 ± 0.11	1.16 ± 0.09	0.39 ± 0.02
10	28	M	2.23 ± 0.11	1.47 ± 0.11	7.94 ± 0.49
11	20	M	3.54 ± 0.18	6.61 ± 0.50	10.9 ± 0.67
12	21	M	11.4 ± 0.06	2.25 ± 0.17	6.48 ± 0.40
13	NDA	NDA	0.97 ± 0.04	0.58 ± 0.04	1.74 ± 0.11
14	21	M	0.64 ± 0.03	< LOQ	0.99 ± 0.06
15	24	F	0.13 ± 0.01	< LOQ	0.55 ± 0.03
16	23	M	1.94 ± 0.10	2.25 ± 0.17	3.67 ± 0.22
17	19	M	2.97 ± 0.16	1.28 ± 0.10	0.79 ± 0.05
18	24	M	1.58 ± 0.09	0.77 ± 0.06	1.99 ± 0.12
19	30	F	0.88 ± 0.03	< LOQ	< LOQ
20	24	F	2.71 ± 0.14	3.47 ± 0.26	7.94 ± 0.49
21	24	M	1.94 ± 0.10	0.57 ± 0.04	0.92 ± 0.06
22	17	F	1.22 ± 0.06	2.99 ± 0.22	2.25 ± 0.14
23	22	M	3.41 ± 0.18	1.44 ± 0.11	6.87 ± 0.42
24	NDA	M	1.69 ± 0.08	0.82 ± 0.06	0.97 ± 0.06
25	27	M	2.08 ± 0.10	3.47 ± 0.26	6.11 ± 0.37
26	27	M	2.51 ± 0.12	2.22 ± 0.17	4.77 ± 0.29
27	25	M	1.11 ± 0.06	0.47 ± 0.04	2.79 ± 0.17
28	22	M	1.97 ± 0.10	3.64 ± 0.27	2.89 ± 0.18
29	17	M	0.75 ± 0.03	< LOQ	1.47 ± 0.09
30	23	M	0.09 ± 0.004	< LOQ	0.97 s± 0.06

*M*  male,* F* female, *SD* standard deviation, *LOQ* limit of quantification, *NDA* no data available

According to Sharma et al. [22], the maximum THC, 11-OH-THC and 11-COOH-THC concentrations in plasma are observed approximately 8, 15 and 81 min, respectively, after onset of smoking. The THC concentration rapidly decreases to 1–4 ng/mL within 3–4 h; however, its residual level is maintained in the body for a long time. The half-life of THC is 1.3 days for infrequent and 5–13 days for frequent users. After smoking a cigarette with 16–34 mg of THC, its main metabolite 11-COOH-THC is detectable in plasma for 2–7 days [22]. The data presented in Table 3 shows the blood levels of THC and its metabolites obtained from 30 DUID cases using the present method. Although in cases 3, 8, 9, 12, 17, 19, 21 and 24, relatively high THC concentrations in whole blood were observed, the concentrations of metabolites were found to be lower. This may lead to the conclusion that the blood samples were taken from examined persons within a short time after cannabis consumption. Conversely, the low concentration of THC in the whole blood sample and high levels of the metabolites indicate that the test samples were taken relatively long after the consumption of cannabis. According to European legal norms [1], ten of the thirty tested subjects were under the influence of THC.

## Conclusions

The analysis of drugs and their metabolites in biofluids usually demands the application of a sample preparation method that allows for full isolation of the analyzed substances from matrices. This is also true for the determination of THC and its metabolites in whole blood samples. Based on results from the present paper, QuEChERS extraction followed by GC–MS/MS represents a convenient and promising method for the analysis of THC, 11-OH-THC and 11-COOH-THC in whole blood. Because of the significant number of experimental variables influencing the QuEChERS analyte recoveries, the application of deuterated internal standards is advisable. Nevertheless, the described method is characterized by good linearity, very low detection limits and satisfactory inter- and intraday precisions for THC, 11-OH-THC and 11-COOH-THC. These features suggest that the presented method is suitable for the routine analysis of THC and its metabolites in whole blood samples and other bodily fluids in living and deceased humans.
